# An Internet of Things Buttons to Measure and Respond to Restroom Cleanliness in a Hospital Setting: Descriptive Study

**DOI:** 10.2196/13588

**Published:** 2019-06-19

**Authors:** Peter R Chai, Haipeng Zhang, Guruprasad D Jambaulikar, Edward W Boyer, Labina Shrestha, Loay Kitmitto, Paige G Wickner, Hojjat Salmasian, Adam B Landman

**Affiliations:** 1 Department of Emergency Medicine Brigham and Women's Hospital Boston, MA United States; 2 The Fenway Institute Boston, MA United States; 3 Harvard Medical School Boston, MA United States; 4 Digital Innovation Hub Brigham and Women's Hospital Boston, MA United States; 5 Department of Psychosocial Oncology and Palliative Care Dana Farber Cancer Institute Boston, MA United States; 6 Environmental Services Brigham and Women's Hospital Boston, MA United States; 7 Department of Quality and Safety Brigham and Women's Hospital Boston, MA United States

**Keywords:** operations research, wireless technology, hygiene, toilet facilities, workflow

## Abstract

**Background:**

Restroom cleanliness is an important factor in hospital quality. Due to its dynamic process, it can be difficult to detect the presence of dirty restrooms that need to be cleaned. Using an Internet of Things (IoT) button can permit users to designate restrooms that need cleaning and in turn, allow prompt response from housekeeping to maintain real-time restroom cleanliness.

**Objective:**

This study aimed to describe the deployment of an IoT button–based notification system to measure hospital restroom cleanliness reporting system usage and qualitative feedback from housekeeping staff on IoT button use.

**Methods:**

We deployed IoT buttons in 16 hospital restrooms. Over an 8-month period, housekeeping staff received real-time notifications and responded to button presses for restroom cleaning. All button presses were recorded. We reported average button usage by hospital area, time of day, and day of week. We also conducted interviews with housekeeping supervisors and staff to understand their acceptance of and experience with the system.

**Results:**

Over 8 months, 1920 requests to clean restrooms in the main hospital lobby and satellite buildings were received. The hospital lobby IoT buttons received over half (N=1055, 55%) of requests for cleaning. Most requests occurred in afternoon hours from 3 PM to midnight. Requests for cleaning remained stable throughout the work week with fewer requests occurring over weekends. IoT button use was sustained throughout the study period. Interviews with housekeeping supervisors and staff demonstrated acceptance of the IoT buttons; actual use was centered around asynchronous communication between supervisors and staff in response to requests to clean restrooms.

**Conclusions:**

An IoT button system is a feasible method to generate on-demand request for restroom cleaning that is easy to deploy and that users will consistently engage with. Data from this system have the potential to enable responsive scheduling for restroom service and anticipate periods of high restroom utilization in a hospital.

## Introduction

### Background

Cleanliness of restrooms frequently serves as a gauge of an organization’s ability to maintain cleanliness throughout all of its facilities. The maintenance of restroom cleanliness is important for 3 reasons. First, dirty restrooms are frequently cited as a source of complaints in many industries. In a 2010 poll, individuals identified dirty restrooms as the top reason to avoid a restaurant with the most common complaints being about clogged toilets, foul odors, out-of-stock supplies, and broken soap or paper dispensers [1,2]. Hospitals are also frequently cited as a source of dirty restrooms; a 2004 survey of 86,000 patients in the United Kingdom showed that only 48% of individuals thought their hospital restrooms were clean [3]. Second, the cleanliness of hospital restrooms is associated with the quality of hospital. In national focus groups designed to inform the Hospital Consumer Assessments of Healthcare Providers and Systems Hospital Survey, 15 of 16 focus groups identified cleanliness of restrooms as a gauge through which participants would measure hospital quality [4]. Similarly, focus groups in Greece demonstrated that patients use cleanliness of restrooms as an indicator of overall quality of the hospital [5]. Third, in health care facilities where nosocomial infections continue to be of a great concern, dirty restrooms can be a source of bacterial pathogens from biowaste. Toilets left unclean may lead to aerosolization of biowaste after flushing, serving as a potential source of infection, especially in immunocompromized individuals [6-8].

### Maintaining Restroom Cleanliness in the Hospital Setting

Hospitals utilize various systems to maintain restroom cleanliness from scheduled, periodic cleaning of high-traffic restrooms to on-demand cleaning based on user feedback. These strategies are symbiotic—ideally, a restroom is cleaned on a schedule based on the number of user requests. Restroom users may request restroom cleaning by notifying nearby staff or calling a posted phone extension to report a dirty restroom. Novel digital techniques to deliver just-in-time notification of restroom cleanliness may use smartphones to report dirty restrooms via a quick response code (QR code), short message service (SMS) short code, or a dedicated app. Digital solutions have the advantage of automating the process of data collection into a central database that can be queried and analyzed to anticipate staffing needs or times when restrooms are most dirty. Yet, existing digital solutions still require user input (eg, using an individual’s smartphone to access an app or to scan a QR code), or require programming of an app, which may be barriers to adoption of such technologies.

An Internet of Things (IoT) button may serve as a simpler, acceptable method to deliver just-in-time notifications of dirty restrooms [9]. An IoT button is a small, electronic device that can be programmed to deliver a customized message to the end user via a wireless network when pressed. In this study, we describe the feasibility and acceptability of deploying an IoT button-based notification system to measure restroom cleanliness in our hospital. We also report descriptive statistics on the use of IoT buttons and share qualitative feedback from housekeeping supervisors and staff on use of IoT buttons.

## Methods

### Overview

We utilized a modified IoT button (GoButton, Visybl Inc, Germantown, MD, USA) configured to deliver just-in-time messages to the housekeeping staff regarding restroom cleaning requests and grounded our investigation in a plan-do-study-act model [10,11]. We conducted our project at a large, urban, academic quaternary care hospital with 763 beds. Our hospital receives approximately 30,000 visitors daily and emergency department (ED) has an annual volume of 60,000 patients.

Before the implementation of the IoT buttons, our housekeeping staff used to clean designated restrooms on a schedule that reflected estimated daily restroom use. For example, our highest volume restrooms in the main lobby have a dedicated housekeeper who inspects the restroom on an hourly basis, whereas an inpatient floor restroom is cleaned once daily.

**Figure 1:**
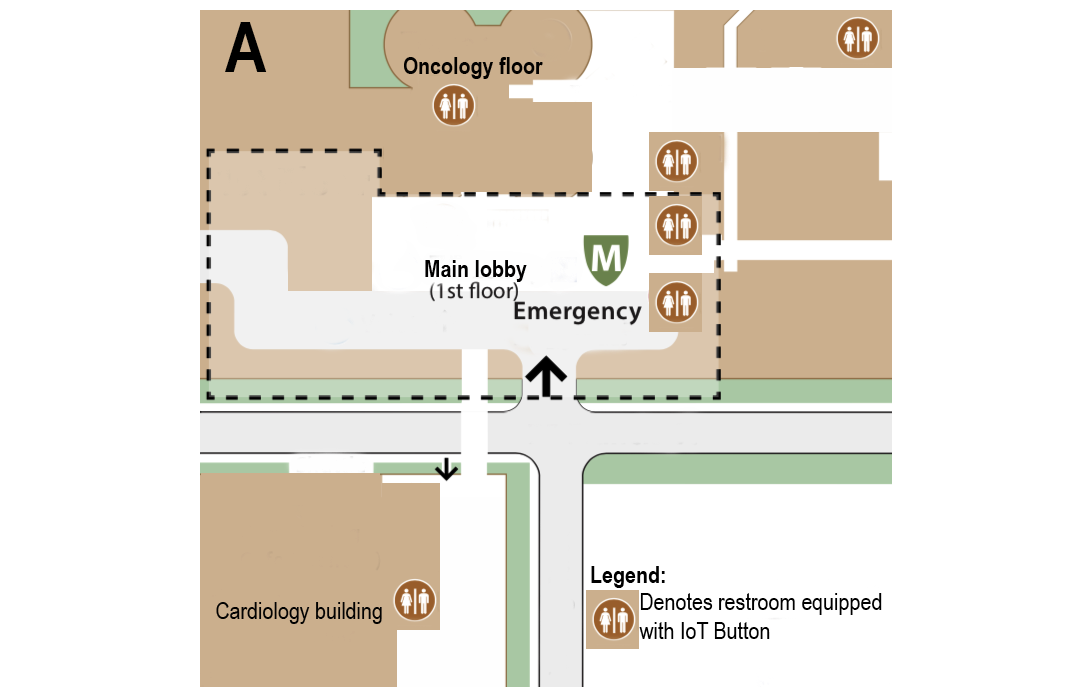
Floor map of major sections of the main hospital building with designated restrooms where Internet of Things buttons were displayed.

We initially selected 9 high-traffic public restrooms throughout the hospital in collaboration with housekeeping leadership (Figure 1). Selection criteria were based on the level of foot traffic and historical high utilization of housekeeping services resources in these restrooms. We also selected the public restrooms on the oncology floor as restroom cleanliness on this floor had previously been a priority for housekeeping staff due to the presence of immunocompromized patients. After 1 month at the request of ED and housekeeping leadership, we also equipped each restroom in the ED (a total of 7) with the IoT button because the cleanliness of these restrooms was identified as an important contributor to patient satisfaction in our hospital. Overall, we included 16 restrooms in our data analysis.

### Internet of Things Button Programming and Deployment

**Figure 2:**
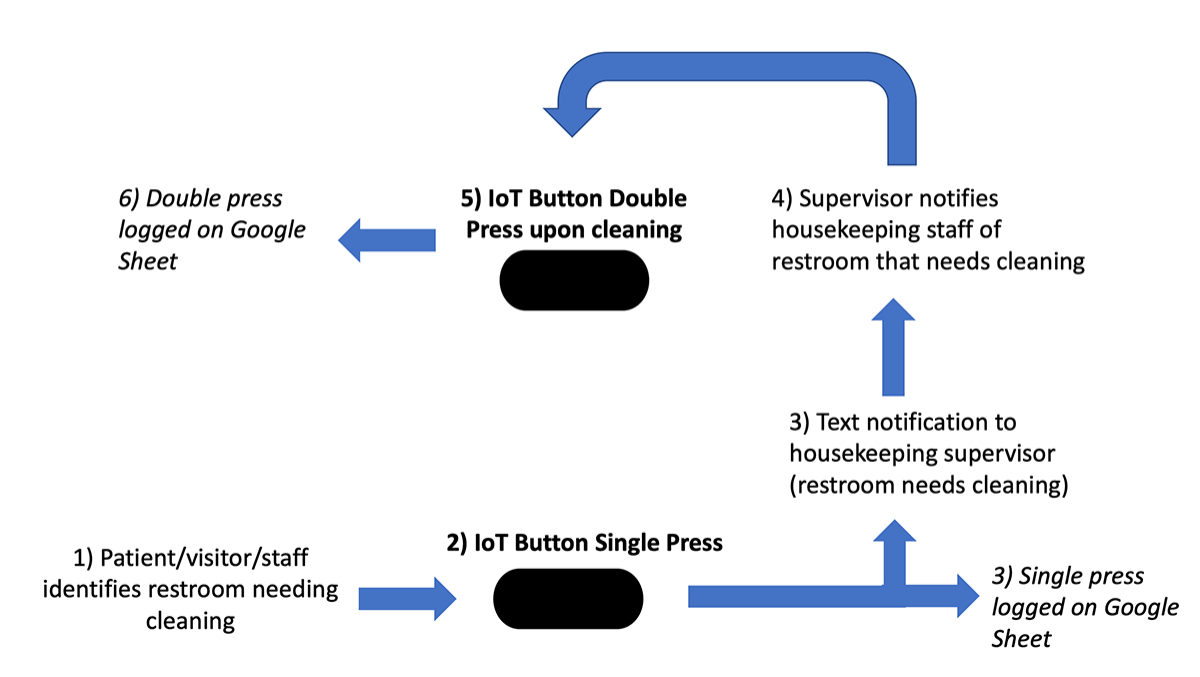
Process diagram of Internet of Things button activation and response.

We mapped each IoT button to a single restroom using the vendor’s Web-based administrator interface [9]. We also used this system to program the IoT button to deliver notifications via SMS messages to housekeeping supervisors based on specific button actions (Figure 2). We designated a single press as a notification that restrooms needed cleaning. In addition, a double press (2 presses of the IoT button within 1s of each other) delivered a notification that the restroom was cleaned by housekeeping staff. Each action—single or double press—was automatically logged on a cloud-hosted, shared spreadsheet. As our housekeeping standard is to respond and clean a restroom within 30 min of a request, we created a button lockout period of 30 min where only the first single press during a 30-min period would deliver a message to housekeeping supervisors. Single presses during the 30-min lockout period were still logged by the interface.

We conducted periodic in-service training for housekeeping staff using a demonstration IoT button. Housekeeping supervisors introduced the device and its intended use to housekeeping staff at daily morning staff meetings. We also conducted in-service training in the evening for housekeepers working the overnight shift. After demonstration of the IoT button, we allowed housekeeping staff to inspect and use the demonstration button. The demonstration button was kept in the housekeeping supervisor’s office so that housekeeping staff could readily access and retrain as needed.

**Figure 3:**
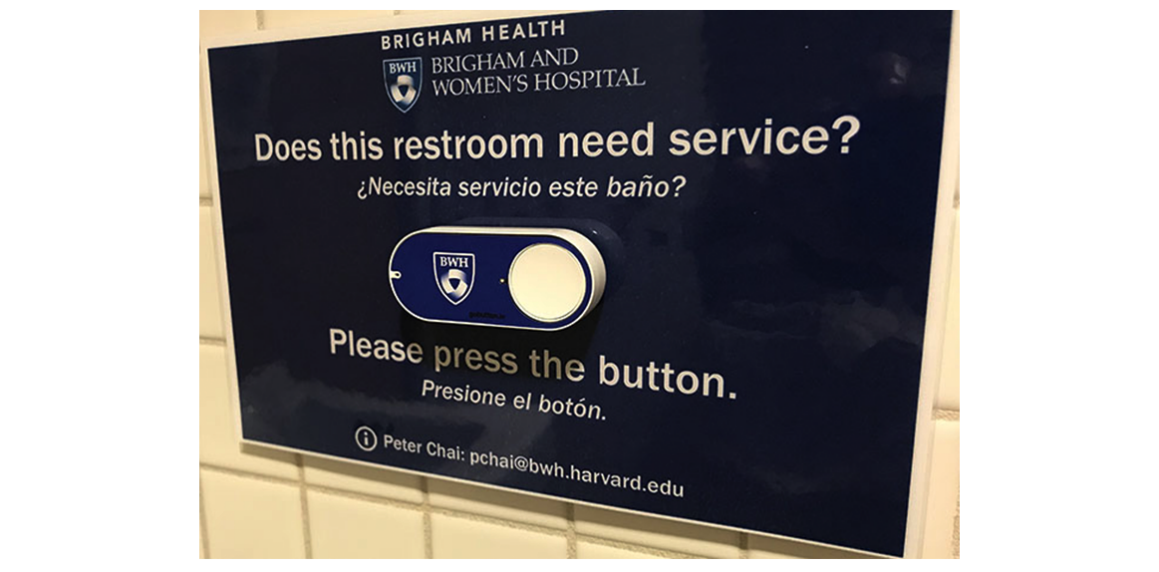
Signage around Internet of Things buttons instructing the public to press the button if they think the restroom needs cleaning.

IoT buttons were mounted in readily accessible areas on restroom walls with public facing signage asking users to press the IoT button once if the restroom needed service (Figure 3). Each button press delivers an SMS message to the designated housekeeping supervisor responsible for restrooms in a specific region who would then dispatch housekeeping staff to assess and clean the restroom. Housekeeping staff were trained by the supervisors to double press the IoT button each time they cleaned the restroom in routine cleanings as well in response to button requests. Periodic retraining occurred during onboarding of new housekeeping staff and during daily housekeeping meetings.

At the end of the study period, we conducted face-to-face group interviews grounded in the technology acceptance model with 4 housekeeping supervisors and 2 staff regarding their experience using the IoT button system [12]. Housekeeping staff and supervisors were recruited during regular operational staff meetings. We utilized a standard interview guide aimed at the intended use of the IoT button, experience integrating the IoT button into the existing restroom cleaning workflow, and optimization of the IoT button for future use. The deployment of the IoT buttons and analysis of the log data was a quality improvement initiative, not human subjects’ research. For the qualitative interview component of the investigation, we obtained institutional review board exemption.

### Data Analysis

We analyzed the audit logs of the IoT buttons, which we obtained from the cloud-based, shared spreadsheet. Audit logs contained the coded location of the IoT button, and the date and time as well as type (single or double press) of the button press. We aggregated all single and double button presses by restroom and also region of the hospital corresponding to the regions of each housekeeping supervisor. We excluded button presses that originated from one IoT button designated as a training device for housekeeping staff. Next, we separated button presses according to housekeeping shifts (7 am to 4 pm , 3 pm to 12 midnight, and 11 pm to 8 am). Presses that occurred during the overlapping shift periods (3 pm to 4 pm, 11 pm to 12 midnight, and 7 am to 8 am) were accounted for in the earlier shift. For example, if a button was activated at 3:30 PM, we counted it as an activation during the 7 am to 4 pm shift. We measured trends of button presses across days of the week. We also calculated a time to next request defined as the number of hours between a single press and a subsequent single press of the same IoT button, as a proxy for how long it took for the bathroom to need cleaning again. We analyzed whether the time to next request was different when cleaning occurred after a request (as evident by a double press of the same IoT button in between the 2 single presses). Data were summarized and reported as mean (SD) and comparisons were made using standard parametric tests (t test and Chi-squared); we used R version 3.6.0 (The R Foundation, Vienna, Austria) to conduct the analyses [13].

Group interviews were conducted by the study staff. Notes regarding participant responses were taken in real time and findings were discussed by members of the study team. Major themes from these discussions were recorded and presented to the group for review. The purpose of qualitative interviews was to gather formative data regarding the early stage deployment of the IoT Button. As a result, we did not complete a formal applied thematic analysis.

## Results

### Overview

**Figure 4:**
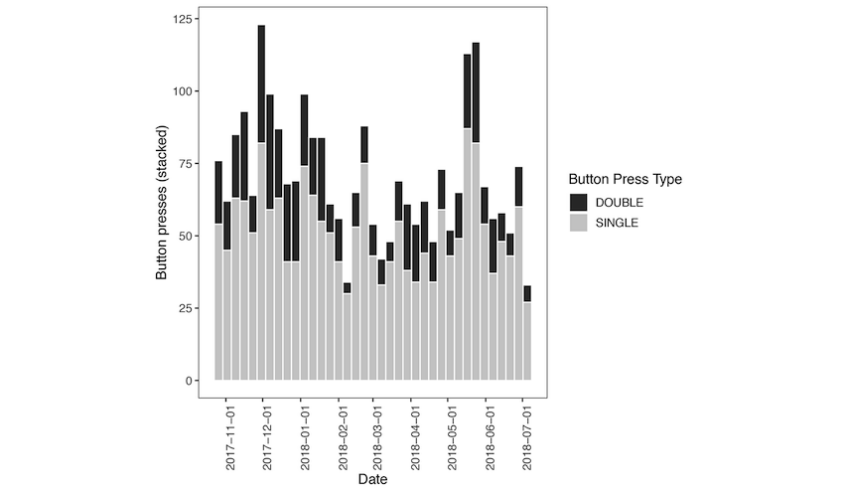
Trends of Internet of Things button utilization stratified by type of press. Each column represents 1 week.

We collected data from 16 restroom IoT buttons from November 2017 to July 2018 (Table 1). Overall, we recorded a total of 2678 presses. We excluded 70 recorded IoT button presses, which were used during housekeeping staff training. A total of 1920 single-press requests for cleaning were recorded, whereas 688 cleaning confirmations (double-press events) were recorded. We observed persistent use of IoT buttons during the study period (Figure 4). During the course of the study period, we had to replace 3 IoT buttons in 3 distinct restrooms that were reported lost. We noted on examination that these buttons likely fell off the signage due to lost integrity of the adhesive originally used. This did not impact our analyses, as we reused the previous button identifiers.

We recorded 1055 total requests for cleaning in the main hospital (ie, men and women’s restrooms in the main entrance lobby of the hospital), accounting for 55% of all requests recorded during the study. The main hospital lobby restrooms were also responsible for the highest number of confirmed cleanings in the main hospital (393/688, 57.1%). On average, 263 button presses were recorded from buttons in the main hospital during the study period (Table 1). There were 384 requests to clean these restrooms in our cardiology building connected to the main hospital over the course of the study period, accounting for 20% of total requests, and a total of 86 confirmed cleaning episodes (86/688, 12.5%). The oncology floor comprised the fewest requests for cleaning in our sample with only 132 (7%) requests during the study period and 22 confirmed cleaning episodes (3%).

**Table 1:** Internet of Things button utilization by hospital location over the study period (8 months).

Location	Number of buttons deployed	Average single presses per button (SD)	Average double presses per button (SD)
Main hospital	4	263.8 (179.8)	97.3 (51.0)
Cardiology building	4	96.0 (43.0)	21.5 (5.4)
Oncology floor	1	132.0 (0)	22.0 (0)
Emergency department	7	49.9 (26.1)	31.1 (14.1)
Overall	16	120.0 (22.8)	45.9 (41.5)

We equipped restrooms in the ED and ED waiting room with IoT buttons 1 month after the start of the study. Despite a late deployment, we recorded an average of 49.9 requests for cleaning in the ED over the study period. There was a total of 349 requests for cleaning in the ED during the study period. Of requests in the ED, 28.7% (100/349) originated from the waiting room. There was a total of 197 confirmed cleans within the ED, 85 of which occurred in the waiting room.

Next, we grouped requests for cleaning by shift (Table 2). An average of 120 requests for cleaning occurred per month during the afternoon shift (3 PM to midnight). There was no variation in requests for cleaning during the work week, although there were fewer requests for cleaning during the weekend, defined as Saturday to Sunday (Table 3).

**Figure 5:**
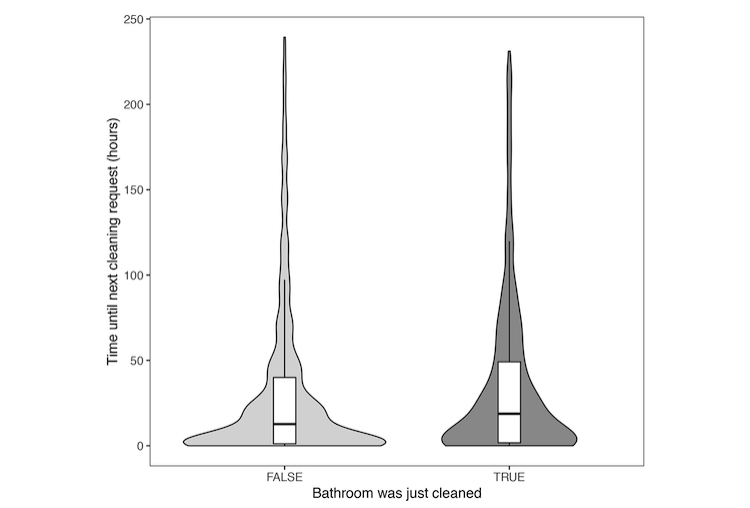
Violin plot demonstrating relationship between an Internet of Things button single press (request for cleaning) and double press (confirmed cleaning request). When confirmed cleaning requests occurred in between two requests for cleaning, the time was longer for a subsequent request. True includes bathrooms with a logged (completed) cleaning event.

The median time between 2 single-press events of the same IoT button was 15.2 hours (interquartile range 45.5 hours; Figure 5). This time was longer when a cleaning event happened in between 2 single-press events (difference 57 min; 95% CI 9-138 min; *P*=.008). We also measured IoT button use over time. Outside of an increase in presses 1 month after deployment with the addition of IoT buttons in the ED, we observed a steady number of button presses per month.

**Table 2:** Average Internet of Things button utilization per month by shift time.

Shift time	Single press, mean (SD)	Double press, mean (SD)
Morning (7 am to 4 pm)	38.9 (18.6)	23.7 (14.9)
Afternoon (3 pm to 12 midnight)	120.0 (53.9)	37.7 (22.9)
Evening (11 pm to 8 am)	33.1 (13.5)	7.4 (5.5)

**Table 3:** Average Internet of Things button utilization by day of the week.

Day of the week	Single press, mean (SD)	Double press, mean (SD)
Monday	29.8 (14.3)	13.4 (7.0)
Tuesday	37.6 (19.5)	10.7 (4.3)
Wednesday	32.9 (19.7)	10.8 (3.9)
Thursday	32.3 (16.8)	9.5 (6.7)
Friday	30.2 (13.9)	11.4 (6.7)
Saturday	13.0 (6.7)	9.3 (10.0)
Sunday	13.9 (5.3)	9.6 (10.3)

### Qualitative Response to Internet of Things Button Deployment

We conducted 2 group interviews; one group consisted of 4 housekeeping supervisors and the other group consisted of 2 housekeeping staff. Our environmental services department employs approximately 10 supervisors. Both groups reported high acceptance of the IoT button to gauge the cleanliness of restrooms. Regarding intention to use, supervisors reported that they wanted to use the IoT button to understand when the volume of requests for cleaning was the highest to dispatch housekeeping staff to clean. In addition, supervisors reported that they intended to use the system to understand workflow of their staff. By recording the double presses, supervisors felt like they were being notified that the restroom was cleaned. In actual use, supervisors reported that they recognized the inherent fallacies of the IoT button system—that there was a lack of information as to why the button was being pressed. Supervisors evolved to respond not to individual requests for clean, but requests in aggregate over time. For example, one supervisor reported they received requests for clean on their phone during the day, and if the frequency of requests started to increase, they would call housekeeping staff and direct them to inspect and clean the restroom. Finally, supervisors reported over the course of the study period, they came to rely on the IoT button system to dispatch the housekeeping staff to potential dirty restrooms.

Housekeeping staff were also accepting of the IoT button system. Staff reported that they felt like most requests for cleaning were likely due to public curiosity with the device. When responding to requests for cleaning, staff reported that there was no context to what was dirty in the restroom. This resulted in staff inspecting and cleaning all aspects of a restroom when a supervisor called. Despite this, staff reported that they did not mind being called by supervisors in response to cleaning requests—staff reported that they felt it was their job to clean restrooms, and if they arrived at a restroom that was reported dirty but found to be clean, then their task would be complete. Housekeeping staff also noted that they frequently forgot to double press the button to indicate that they cleaned the restroom because it represented an additional step in their workflow, but if their supervisor had called and dispatched them to clean the restroom, they would double press the IoT button to show supervisors that their job was complete. Overall, housekeeping staff reported acceptance of the IoT button system during the study period and reported that the system was a tool that helped them keep restrooms cleaner.

## Discussion

### Principal Findings

Our data demonstrate that an IoT button is a feasible method to gauge the public perception of restroom cleanliness in a hospital. Over the course of 1 year, we demonstrated persistent use of the IoT button system. Housekeeping supervisors and staff reported that real-time data from the IoT button were helpful in maintaining the cleanliness of restroom. By the end of the investigation, both staff and supervisors had integrated the IoT button system into their daily workflow demonstrating its adoptability. In addition, we only had to replace 3 IoT buttons during the study period—these buttons were lost likely due to degradation of adhesive that was used to affix the buttons to placards. These results are important because they demonstrate that a low-cost, IoT button solution can be deployed at a large hospital setting, the public will continue to engage with the system, and meaningful, actionable data can be generated to assist housekeeping with daily services.

The IoT button system allowed us to measure restroom cleaning requests in a dynamic manner. Due to the simplicity of the device, we experienced more requests from individuals using the restrooms instead of confirmation of cleaning events by housekeeping staff; 71.29% (1920/2693) of presses of the button came from requests for a restroom to be cleaned. Restrooms in places with higher traffic such as those in the main hospital received higher requests for cleaning in comparison with restrooms on an inpatient ward. These findings suggest that the IoT button system can enable contextual awareness of restroom cleanliness to enable real-time housekeeping staffing levels based on requests for cleaning [14]. For example, during times of high public traffic, if there is a real-time increase in requests for clean in one part of the hospital, this could be an indication to housekeeping leadership to move a housekeeper to that area from one where there are less requests for clean. In this manner, we think that future iterations of the IoT button system can help guide and even predict how to efficiently deploy housekeeping staff in the hospital.

Of note, the IoT button data indicated that when a restroom was confirmed cleaned by housekeeping staff it would take longer for a subsequent cleaning request to be requested at that restroom. Although the observed difference was only 57 min, it should be noted that many restrooms are already cleaned multiple times a day, and a larger difference in the data may be practically infeasible. In addition, staff reported that if they cleaned restrooms based on their set schedule, they frequently did not log these cleaning episodes on the IoT button system. With increased adherence to logging cleaning episodes on the IoT button system, this system could be used to understand response times to requests for cleaning. More frequent in-service training of staff members may help improve reporting of cleaning episodes. The IoT button system may also help not only respond to dirty restrooms, but over time, maintain cleaner restrooms, and improve perceived quality of a hospital [15]. This analysis is likely limited by the fact that less than 2 cleaning events were recorded using a double press for 96% of location-days. With improved training of staff and more accurate recording of double presses, the IoT button system may be used to maintain cleaner restrooms.

Our formative qualitative data support end-user adoption and integration of the IoT button into the daily workflow of our housekeeping staff, despite its introduction as a quality improvement project. Major identified themes surrounded the ability to access and transmit restroom data in real time, and the use of the button to report cleaning episodes. Importantly, housekeeping staff were willing and able to double press the IoT button as they responded to requests by their supervisors to clean restrooms demonstrating the importance of completing feedback loops and reinforcing their daily task of helping to maintain clean restrooms [16]. Despite identified limitations with the workflow and data, supervisors continued to utilize the IoT button to understand the pulse of restroom cleanliness during the day whereas housekeeping staff found the IoT button an easy method in which to communicate completion of a task (cleaned restroom) to supervisors.

### Challenges in Implementing the Internet of Things Button System

We experienced data security issues as we considered scaling the IoT button system. The IoT button version we used only supports Wi-Fi Protected Access 2 (WPA2) encryption, which only requires a single preshared key (PSK) to connect to a network. Learning this single PSK could lead to system compromise, and potential alteration of restroom data [17]. Note that the Wi-Fi network used for the IoT buttons was separate from the network used for clinical care. Future iterations of IoT buttons can address this by enabling access to WPA2 Enterprise (also known as 802.11i) that requires a unique username and password and a preinstall unique encryption key, thereby providing additional security, or utilizing onboard cellular networks further isolating the IoT buttons from the hospital network and preventing a system downtime in the event of a Wi-Fi failure [9].

We also experienced human factor challenges in implementation of the IoT button. There are a large number of housekeepers who work in our hospital, and it was difficult to maintain housekeeper adherence to double press the button each time they cleaned a restroom or responded to a request for clean. Our qualitative data reflect that this was an additional step in their workflow; this likely led to a decreased number of double presses confirming cleaning by housekeeping. We therefore were unable to accurately calculate a response time from a request for clean to the time that a housekeeper actually cleaned the restroom. We believe that with continued deployment of the IoT button in our hospital, the process of double presses as the restroom is cleaned will become part of the housekeeping workflow leading to more robust data. Some other potential alternatives to relying upon housekeepers to double press the IoT button include deploying an integrated IoT button and radiofrequency identification (RFID) placard to allow housekeepers to scan their badges when their cleaning is complete, or RFID badges that log the movement of housekeepers into restrooms. This alternative technique will require user acceptability evaluation before the deployment in conjunction with an IoT button system. Despite these challenges, we believe that the IoT button system was able to provide valuable data regarding the pulse of restroom cleanliness in our hospital. During the study period, our housekeeping supervisors perceived the IoT button system as a key part of their daily workflow.

### Limitations

This study had several limitations. First, we lacked contextual data surrounding IoT button presses. We do not know if users pressed the button because restrooms were dirty, or in need of service (eg, replenishing supplies and unclogging drains). In addition, although we were able to record requests for cleaning, we do not know what aspect of the restroom was dirty. Future studies could utilize an in-person monitor to describe reasons for a request for clean and further understand the patterns of the IoT button use. Second, despite acceptability of the IoT button system, we continued to have some human factors issues regarding adoption of a double press to log a completed cleaning procedure of restrooms. We therefore may have underestimated the number of responses to clean restrooms using our system. Third, our qualitative analysis was limited by sample size, and our future research includes an expanded and more rigorous qualitative evaluation on the adoption and sustainability of IoT buttons as part of the housekeeping workflow. Finally, from a technological perspective, the IoT buttons only have a battery life to 2000 presses. A cost analysis of the benefits of replacing IoT buttons in comparison to potential improvements in patient satisfaction may be required to assess the long-term sustainability and value of this technology.

### Conclusions

Overall, this study demonstrates that we were able to deploy an IoT button system that measures restroom cleanliness and can also be used by internal staff to log activities related to servicing restrooms. Although there are a variety of technologies that can be used to deliver similar just-in-time notifications regarding restroom cleanliness, we found our system easy to deploy, and engaging. Even 1 year after deployment of the system, we continued to receive notifications through the IoT button for cleaning requests. We additionally were able to understand some potential trends in the ebbs and flows of service request throughout the workday and workweek. We anticipate that with continued optimization of the IoT button system, the data generated could be used to inform alternative staffing models that respond to real-time changes in the need for housekeeping services in a hospital system.

## Abbreviations

ED: emergency department
IoT: Internet of Things
PSK: preshared key
QR code: quick response code
RFID: radiofrequency identification
SMS: short message service
WPA2: Wi-Fi Protected Access 2
